# COVID-19 Among Patients With Inflammatory Rheumatic Diseases

**DOI:** 10.3389/fimmu.2021.651715

**Published:** 2021-04-16

**Authors:** Sinem Nihal Esatoglu, Koray Tascilar, Hakan Babaoğlu, Cemal Bes, Berna Yurttas, Servet Akar, Ozlem Pehlivan, Cansu Akleylek, Duygu Tecer, Emire Seyahi, Tuba Yuce-Inel, Nilufer Alpay-Kanitez, Erdal Bodakci, Emre Tekgoz, Seda Colak, Ertugrul Cagri Bolek, Suleyman Serdar Koca, Umut Kalyoncu, Ozan Cemal Icacan, Serdal Ugurlu, Hande Ece Oz, Vedat Hamuryudan, Gulen Hatemi, the Turkish Society for Rheumatology COVID-19 Registry Investigators, Ayse Cefle

**Affiliations:** ^1^ Department of Rheumatology, Gaziosmanpasa Research and Training Hospital, Health Sciences University, Istanbul, Turkey; ^2^ Department of Internal Medicine 3 - Rheumatology and Immunology, Friedrich-Alexander University Erlangen- Nuremberg and Universitätsklinikum Erlangen, Erlangen, Germany; ^3^ Division of Rheumatology, Department of Internal Medicine, Faculty of Medicine, Gazi University, Ankara, Turkey; ^4^ Department of Rheumatology, Sadi Konuk Education and Research Hospital, Health Sciences University, Istanbul, Turkey; ^5^ Division of Rheumatology, Department of Internal Medicine, Cerrahpasa Faculty of Medicine, Istanbul University−Cerrahpasa, Istanbul, Turkey; ^6^ Division of Rheumatology, Department of Internal Medicine, Faculty of Medicine, Izmir Katip Celebi University, Izmir, Turkey; ^7^ Department of Rheumatology, Umraniye Training and Research Hospital, Istanbul, Turkey; ^8^ Division of Rheumatology, Department of Internal Medicine, Faculty of Medicine, TC Demiroglu Bilim University, Istanbul, Turkey; ^9^ Department of Rheumatology, Gülhane Faculty of Medicine, Health Sciences University, Ankara, Turkey; ^10^ Division of Rheumatology, Department of Internal Medicine, Faculty of Medicine, Dokuz Eylül University, Izmir, Turkey; ^11^ Division of Rheumatology, Department of Internal Medicine, Faculty of Medicine, Koc University, Istanbul, Turkey; ^12^ Department of Rheumatology, Eskisehir City Hospital, Eskisehir, Turkey; ^13^ Division of Rheumatology, Department of Internal Medicine, Faculty of Medicine, Hacettepe University, Ankara, Turkey; ^14^ Division of Rheumatology, Department of Internal Medicine, School of Medicine, Firat University, Elazig, Turkey

**Keywords:** COVID-19, rheumathoid diseases, SARS CoV-2, DMARDs, biologic DMARDs

## Abstract

**Background:**

The course of novel coronavirus disease 2019 (COVID-19) has been of special concern in patients with inflammatory rheumatic diseases (IRDs) due to the immune dysregulation that may be associated with these diseases and the medications used for IRDs, that may affect innate immune responses.

**Objective:**

In this cohort study, we aimed to report the disease characteristics and variables associated with COVID-19 outcome among Turkish patients with IRDs.

**Methods:**

Between April and June, 2020, 167 adult IRD patients with COVID-19 were registered from 31 centers in 14 cities in Turkey. Disease outcome was classified in 4 categories; (i) outpatient management, (ii) hospitalization without oxygen requirement, (iii) hospitalization with oxygen requirement, and (iv) intensive care unit (ICU) admission or death. Multivariable ordinal logistic regression analysis was conducted to determine variables associated with a worse outcome.

**Results:**

165 patients (mean age: 50 ± 15.6 years, 58.2% female) were included. Twenty-four patients (14.5%) recovered under outpatient management, 141 (85.5%) were hospitalized, 49 (30%) required inpatient oxygen support, 22 (13%) were treated in the ICU (17 received invasive mechanic ventilation) and 16 (10%) died. Glucocorticoid use (OR: 4.53, 95%CI 1.65-12.76), chronic kidney disease (OR: 12.8, 95%CI 2.25-103.5), pulmonary disease (OR: 2.66, 95%CI 1.08-6.61) and obesity (OR: 3.7, 95%CI 1.01-13.87) were associated with a worse outcome. Biologic disease-modifying antirheumatic drugs (DMARDs) do not seem to affect COVID-19 outcome while conventional synthetic DMARDs may have a protective effect (OR: 0.36, 95%CI 0.17-0.75). Estimates for the associations between IRD diagnoses and outcome were inconclusive.

**Conclusions:**

Among IRD patients with COVID-19, comorbidities and glucocorticoid use were associated with a worse outcome, while biologic DMARDs do not seem to be associated with a worse outcome.

## Introduction

The novel coronavirus disease 2019 (COVID-19) caused by severe acute respiratory syndrome coronavirus 2 (SARS-CoV-2) runs a variable clinical course including asymptomatic infection, flu-like symptoms, isolated anosmia or hyposmia, mild to severe pneumonia, involvement of various organs or a cytokine storm, which may eventually prove fatal (1).

All age groups and patient populations can be affected by COVID-19, however, individuals with an older age, and those with co-morbidities have a higher risk for more severe disease (2). Inflammatory rheumatic diseases (IRDs) have especially been a concern for a number of reasons. The immune dysregulation that may be associated with IRDs or by the medications used for these may affect innate immune responses that play a critical role in preventing viral replication and in development of an adaptive immune response. Inability to reduce the viral load in the early stages of the disease may result in an exaggerated inflammatory reaction, leading to tissue damage and multiple organ failure (3). A higher infection rate was observed in patients with IRDs compared to their family members (4) and higher respiratory failure rates were reported in COVID-19 patients with IRDs compared to those without IRDs (5). Medications that are used for IRDs may influence the outcome of COVID-19. Hydroxychloroquine (HQ), chloroquine and baricitinib were suggested to have antiviral effects and patients on these drugs were proposed to be less likely to have severe COVID-19 outcomes due to reduced viral replication during the early phase (6). Moreover, physician-reported COVID-19 Global Rheumatology Alliance (GRA) registry suggested that biologic disease-modifying antirheumatic drugs (bDMARDs), which are used for the treatment of cytokine storm that is responsible for tissue damage and multiple organ failure in COVID-19, may prevent this condition in patients with IRDs who are already using bDMARDs, if infected by SARS-CoV-2 (7).

The Turkish Society for Rheumatology implemented a registry for recording COVID-19 patients with IRDs, with the aim of understanding the characteristics and outcome of COVID-19 among these patients and the association of types and characteristics of IRDs, treatment modalities that were used for IRDs, and co-morbidities with the outcome of COVID-19. In addition to being one of the largest COVID-19 registries among IRD patients, we utilized a novel and more granular outcome assessment in contrast to previous studies of COVID-19 in IRD patients which have focused on a single dichotomous outcome such as hospitalization, severe disease or mortality. We aimed to report the outcome across a spectrum including outpatient management, hospitalization without oxygen requirement, hospitalization with oxygen requirement, intensive care unit (ICU) admission or death. This spectrum was modified and adapted from the WHO clinical progression scale (8). We report here the clinical characteristics and outcomes of the first 165 patients in this registry.

## Materials and Methods

### Study Design and Participants

All members of the Turkish Society for Rheumatology were invited through e-mail. The registry was built and maintained using the Research Electronic Data Capture (REDCap) web application (https://www.project-redcap.org/) (9). The study was approved by the Ethics Committee of Istanbul University–Cerrahpaşa (83045809-604.01.02). The project did not receive funding. This study was conducted in accordance with the Declaration of Helsinki on Ethical Principles. Since this was a retrospective database study, informed patient consent was not required.

Inclusion criteria were a diagnosis of IRD confirmed by a rheumatologist and a diagnosis of COVID-19 by at least one of the following; (i) a positive reverse-transcription polymerase chain reaction (RT-PCR) for SARS-CoV-2 RNA (ii) detection of antibodies against SARS-CoV-2, or (iii) symptoms and computed tomography (CT) findings highly-suggestive of COVID-19. Only adult (≥18 years old) patients were included in the registry. Patients with a presumptive diagnosis solely based on symptoms were excluded.

### Case Report Form

A standard case report form was used for collecting demographic data, primary IRD diagnoses, comorbid conditions, smoking status, history of flu vaccination, medications used for IRDs, dose of glucocorticoids, concomitant drugs deserving special interest regarding COVID-19 including angiotensin-converting enzyme (ACE) inhibitors, angiotensin II receptor blockers (ARBs), non-steroidal anti-inflammatory drugs (NSAIDs), cyclooxygenase-2 (COX-2) inhibitors and phosphodiesterase type 5 inhibitors, presumed route of COVID-19 transmission, infection among household members, interval between symptom onset and diagnosis of COVID-19, diagnostic tests and medications used for COVID-19, requirement of oxygen therapy, intensive care unit admission, complications, disease activity of the rheumatic condition before and after COVID-19, and the outcome of COVID-19.

Patient initials, the last 6 digits of the 11-digit unique national identification number, gender and age of the patients and contact information of the participating physician were obligatory fields. Participants were advised to fill the other fields only if they had reliable information. We contacted participants before the analysis in order to clarify any inconsistencies.

Standard treatment protocols for COVID-19 in Turkey, as advised by the Turkish Ministry of Health (TMoH) are provided in the **Supplementary Material**.

### Outcome of COVID-19

Disease outcome was classified in 4 ordinal categories; (i) outpatient management, (ii hospitalization without oxygen requirement, (iii) hospitalization with oxygen requirement, and (iv) ICU admission or death. The analyses focused on 3 primary questions, namely associations of COVID-19 outcome with the type of primary IRD diagnosis, type of IRD treatment, and comorbidities.

### Statistical Analysis

Continuous data were summarized as mean ± SD or median and IQRs as appropriate. Categorical data were summarized as percentages. We summarized the proportions of primary IRD diagnoses, IRD treatments, and comorbidities for each outcome category using dot charts. For analyses related to the primary questions above we used proportional odds logistic regression where the dependent variable was the 4-level ordinal outcome and the independent variable of interest was the type of IRD treatment, comorbidity or primary IRD diagnosis. IRD treatments were categorized into no treatment, glucocorticoids (any dose, moderate/high dose (≥7.5 mg/day of prednisolone) and low dose), HQ, colchicine, conventional synthetic DMARDs (csDMARDs) (methotrexate, sulfasalazine, leflunomide), immunosuppressives (mycophenolate mofetil, tacrolimus, azathioprine), rituximab and other bDMARDs (tumor necrosis factor inhibitors (TNFis), abatacept, anakinra, secukinumab, tocilizumab). Comorbidities were categorized as no comorbidity, hypertension, diabetes mellitus, cardiovascular disease, pulmonary disease, chronic kidney disease, obesity, cerebrovascular accident, cancer, additional immune disorder including psoriasis, inflammatory bowel disease and renal transplantation, and others. IRD diagnoses were categorized as rheumatoid arthritis (RA), spondyloarthritis (SpA), connective tissue diseases, familial Mediterranean fever (FMF), Behçet syndrome (BS) and other IRDs. Independent variables of interest were analyzed as binary variables i.e. a separate model was fitted for each IRD treatment, each comorbidity and IRD diagnosis category to calculate crude odds ratios and 95% confidence intervals for being in a worse outcome category in comparison to its absence. We used causal models to identify minimal sufficient sets of adjustment covariates separately for IRD treatment models, comorbidity models and primary IRD diagnosis models using DAGitty (10). Causal assumptions for covariate selection are depicted as directed acyclic graphs in the **Supplementary Material (Figures S1-S3)**. Namely, we used different combinations of age, sex, comorbidity, use of solo or combination treatments and smoking status for adjustment. In order to keep model degrees of freedom to the minimum, we dichotomized smoking status as ever vs never and comorbidities as any vs none in adjustments. We additionally performed the same analyses using a 5-level ordinal outcome, classifying ICU and death as separate categories. Missing smoking data was assumed to be missing completely at random and multivariable analysis for comorbidities were carried out on cases with complete data. Analyses were performed using the open-source R software (v. 4.0.1, R Foundation for Statistical Computing, Vienna, Austria).

## Results

Between April 20, 2020 and June 16, 2020, a total of 167 patients were recorded from 31 centers (28 tertiary centers and 3 private hospitals) in 14 cities. Two patients who did not have a confirmed diagnosis of COVID-19 were excluded. The remaining 165 patients were included in the analyses.

### Demographic and Clinical Characteristics

The demographic and clinical characteristics of the patients including smoking status and comorbidities, primary IRD diagnoses and IRD treatments that were used at the time of COVID-19 diagnosis are summarized in **Table 1**. More details on IRD diagnoses and treatments are provided in the **Supplementary Table S1**.

**Table 1:** Demographics, clinical characteristics, IRD diagnoses and rheumatic drugs of IRD patients with COVID-19.

Characteristics	
Age (mean ± SD), years	50 ± 15.6
Age group (n, %)	
≥65 years	34 (21)
<65 years	131 (79)
Female (n, %)	96 (58.2)
Smoking status, n/N, (%)	
Current	19/146 (13)
Past	30/146 (20.5)
Never	97/146 (66.5)
No (%) of comorbidities	
None	80 (48.5)
1	51 (31)
2	15 (9)
3	15 (9)
≥4	4 (2.5)
Comorbidities^a^, n (%)	
Hypertension	51 (31)
Diabetes mellitus	17 (10)
Cardiovascular disease	15 (9)
Chronic kidney disease	6 (4)
Pulmonary disease	23 (14)
Cerebrovascular accident	5 (3)
Malignancy	6 (4)
Obesity	10 (6)
Psoriasis	4 (2)
Other^b^	9 (5)
History of influenza vaccination, n/N, (%)	8/112 (7)
Diagnosis^a^, n (%)	
Rheumatoid arthritis	60 (36)
Spondyloarthritis	42 (25)
Connective tissue disease	29 (18)
Familial Mediterranean fever	14
Behçet syndrome	15 (9)
Vasculitis	6 (4)
Other^c^	12 (7)
Treatment	
NSAIDs, n/N, (%)	
Yes and continued	7/163 (4)
Yes and discontinued	15/163 (9)
COX-2 inhibitors, n/N, (%)	
Yes and discontinued	3/163 (2)
No	160/163 (98)
Prednisolone^d^, n (%)	
None	97 (59)
<7.5 mg/day	49 (30)
≥7.5 mg/day	18 (11)
Colchicine, n (%)	25 (15)
Hydroxychloroquine, n (%)	40 (24)
CsDMARDs, n (%)	79 (48)
Immunosuppressives, n (%)	16 (10)
bDMARDs, n (%)	36 (22)

bDMARDs, biologic DMARDs; COX-2, cyclooxygenase-2; csDMARDs, conventional synthetic DMARDs; DMARDs, disease-modifying antirheumatic drugs; IRD, inflammatory rheumatic disease; NSAIDs, non-steroidal anti-inflammatory drugs.

a Comorbidities and IRD diagnoses are non-mutually-exclusive.

b 2 patients with inflammatory bowel disease, 1 with renal transplantation, 1 with cirrhosis, 1 with pregnancy, 3 with psychiatric disorder, and 1 with neuromuscular disease.

c 3 patients with gout, 3 with adult onset Still’s disease, 2 with polymyalgia rheumatic, 2 with juvenile idiopathic arthritis, 1 with sarcoidosis, and 1 with undifferentiated arthritis.

d Prednisolone dose was unknown in 1 patient.

### Diagnosis of COVID-19

The mean time interval between symptom onset and diagnosis was 4.8 ± 4.5 days. COVID-19 was diagnosed by RT-PCR in 114 patients, by highly-suggestive lung CT scans in 50, and by antibody testing in 1 patient. Among the 114 patients who were RT-PCR positive, 95 (83%) also had highly-suggestive CT findings. Only 13 (8%) patients were asymptomatic prior to diagnosis of COVID-19. In 11 of these patients, the reason for SARS-CoV2 testing was a history of close contact with a COVID-19 patient or a history of visit to a high-risk country. In the remaining 2 patients, SARS-CoV2 pneumonia pattern was detected on CT scans that were performed during their hospitalization for reasons unrelated to COVID-19.

The possible routes of transmission of COVID-19 and the number of COVID-19 diagnosis among household members are provided in the **Supplementary Material**.

### Medications for COVID-19

All but 2 patients used HQ, 129 patients (78%) used azithromycin and 50 patients (30%) used favipiravir. These were in line with the TMoH protocols until August 2020, after which favipiravir use became more common. For patients with severe pneumonia, tocilizumab was used in 7 (4%), intravenous immunoglobulin in 2 (1%), glucocorticoids in 13 (8%) and convalescent plasma in 3 patients (2%). Among the 7 patients who were given antibiotics, bacterial growth was confirmed with cultures in 3 patients, 1 patient had a deep soft tissue infection in a lower extremity and 3 patients had persistently elevated acute phase reactants. Anticoagulation prophylaxis was given to 44 (38%) of the 115 patients with available information regarding anticoagulation **(** **Table 2** **).**

**Table 2:** Management and outcome of COVID-19 in IRD patients.

Characteristics	n (%)
Outcome	
Death	16 (10)
ICU	22 (13)
Hospitalization without oxygen requirement	92 (56)
Hospitalization with oxygen requirement	49 (30)
Outpatient management	24 (14.5)
IRD diagnoses of deceased patients, n/N^a^, (%)	
Rheumatoid arthritis	8/60, (13)
Systemic lupus erythematosus	2/13, (15)
Psoriatic arthritis	1/10, (10)
FMF+SpA	1/(14 FMF, 42 SpA)
Behçet syndrome	1/15, (7)
Polymyalgia rheumatica	1/2
Sarcoidosis	1/1
Goodpasture syndrome	1/1
Complications^b^	
None	134 (81)
ARDS	22 (13)
Bacterial infection	7 (4)
Thromboembolism	2 (1)
Myocarditis	1
ITP associated with SLE	1
Treatment	
HQ	163 (99)
HQ+AZA±Oseltamivir	129 (78)
Lopinavir/ritonavir	5 (3)
Favipiravir	50 (30)
Glucocorticoid	13 (8)
Tocilizumab	7 (4)
Intravenous immunoglobulin	2 (1)
Antibiotics	7 (4)
Convalescent plasma	3 (2)
Anticoagulation	44/115 (38)

ARDS, acute respiratory distress syndrome; AZA, azithromycin; FMF, familial Mediterranean fever; HQ, hydroxychloroquine; ICU, intensive care unit; IRD, inflammatory rheumatic disease; ITP, immune thrombocytopenia; SLE, systemic lupus erythematosus; SpA, spondyloarthritis.

a n/N, Number of deceased patient/s/Total number of patient/s.

b Thirty-three complications occurred in 31 patients.

### Course and Outcome of COVID-19

Among the 165 patients, 24 (14.5%) (mean age: 45.2 ± 12.8 years, 14 (58%) female) had recovered with outpatient management, 141 (85.5%) (mean age: 51.4 ± 15.8 years, 82 (58%) female) were hospitalized, of whom 49 (30%) had required inpatient oxygen support, 27 (16%) (mean age: 58.4 ± 16 years, 16 (59%) female) were treated in the ward, 22 (13%) (mean age: 60 ± 16.5 years, 16 (73%) female) were treated in the ICU and 16 (10%) (mean age: 61 ± 17 years, 12 (75%) female) had died **(** **Supplementary Figure S4** **).** Of the patients treated in the ICU, 17 (10.3%) required invasive mechanic ventilation **(** **Table 2** **).** Among the patients who died, all except 1 were treated in the ICU. The remaining patient was planned to be transferred to the ICU, but died before this was possible. Mortality rate among patients who were admitted to the ICU was 15/22 (68%). Detailed information regarding patients who needed ICU admission and those who died are provided in the **Supplementary Table S2**.

Thirty-three complications occurred in 31 patients (19%). These were acute respiratory distress syndrome in 22 patients, bacterial infection in 7, ischemic stroke, pulmonary thromboembolism, acute heart failure due to myocarditis, and immune thrombocytopenia possibly associated with underlying SLE in 1 patient each **(** **Table 2** **).**

### Association of the Type of IRD Diagnosis, IRD Treatments and Comorbidities With the Outcome of COVID-19

**Figure 1:**
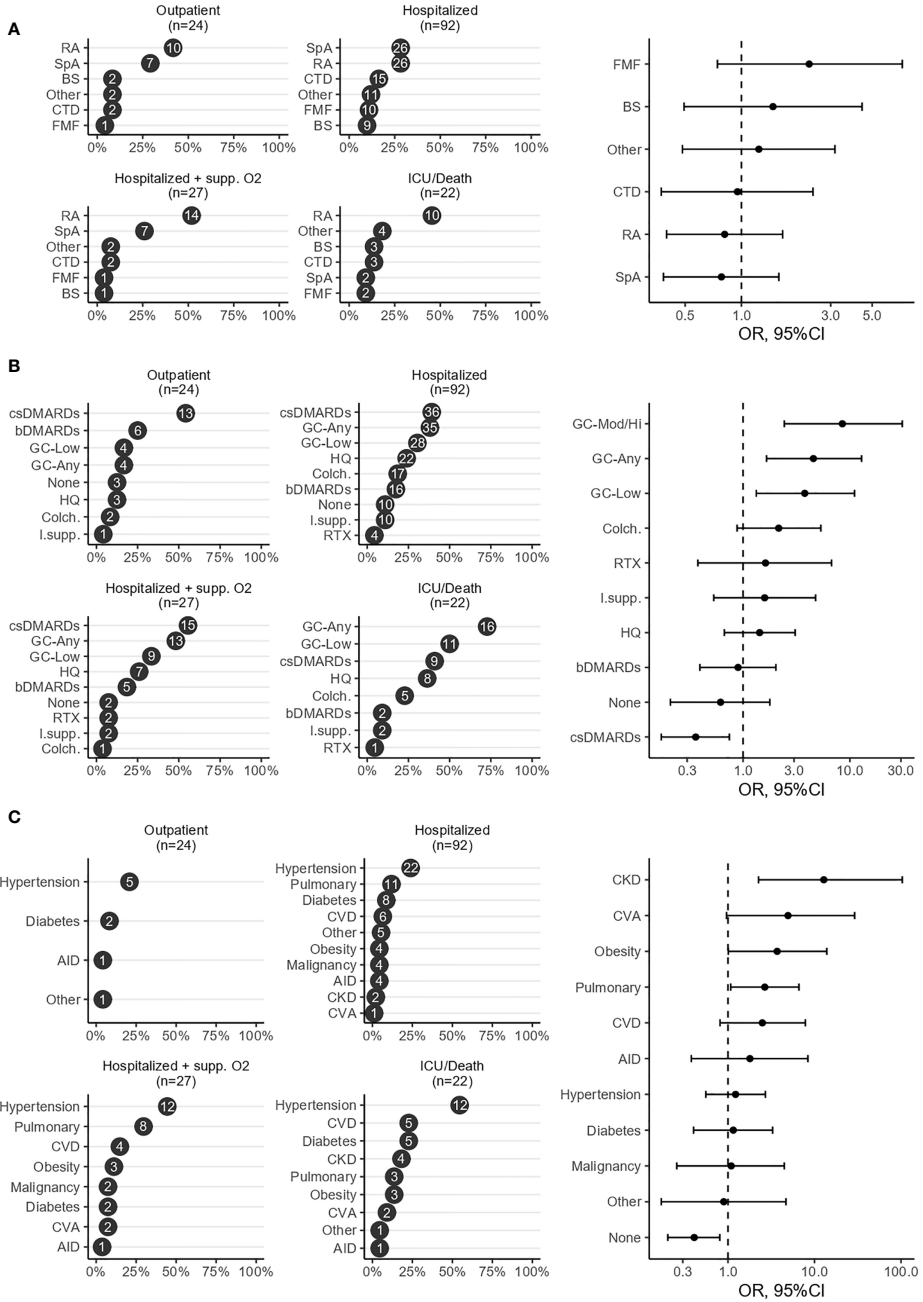
Risk factors of a worse COVID-19 outcome in patients with inflammatory rheumatic diseases. Figure illustrates the ranked proportions of IRD diagnoses **(A)**, IRD treatments **(B)**, and comorbidities **(C)** for each outcome category and the association of each with a worse outcome, analyzed using proportional odds logistic regression where the dependent variable was the 4-level ordinal outcome and the independent variable of interest was the type of IRD treatment, comorbidity or primary IRD diagnosis. High/moderate glucocorticoid dose (GC-Mod/Hi) was defined as a daily dose of≥7.5 mg prednisolone. csDMARDs included methotrexate, sulfasalazine, leflunomide, immunosuppressives included mycophenolate mofetil, tacrolimus, azathioprine and bDMARDs included tumor necrosis factor inhibitors, abatacept, anakinra, secukinumab and tocilizumab. AID, additional immune disorder; bDMARDs, biologic DMARDs; BS, Behçet syndrome; CKD, chronic kidney disease; Colc., colchicine; csDMARDs, conventional synthetic DMARDs; CTD, connective tissue disease; CVA, cerebrovascular accident; CVD, cardiovascular disease; DMARDs, disease-modifying antirheumatic drugs; FMF, Familial Mediterranean fever; GC, glucocorticoid; ICU, intensive care unit; IRD, inflammatory rheumatic disease; I.supp., immunosuppressives; HQ, hydroxychloroquine; RA, rheumatoid arthritis; RTX, rituximab; SpA, spondyloarthritis.

**Figure 1** shows the proportions of IRD diagnoses, IRD treatments, and comorbidities for each outcome category, and the association of each with a worse outcome. Unadjusted estimates suggested a higher risk of worse outcomes for RA and “Other” IRDs, however, after adjustment for age, sex and comorbidity, FMF and BS patients showed the highest relative risk of worse outcomes when the point estimates are considered **(** **Table 3** **).** These estimates however are not sufficiently precise to make any strong conclusions.

**Table 3:** Crude and adjusted Odds Ratios of the association of primary IRD diagnosis, IRD treatment, and comorbidity with a worse COVID-19 outcome.

Variable	OR, Crude (95%CI)	OR, Adjusted (95%CI)
**IRD diagnosis** ^a^		
Familial Mediterranean fever	0.98 (0.35 to 2.73)	2.3 (0.74 to 7.25)
Behçet syndrome	1.03 (0.36 to 2.88)	1.47 (0.49 to 4.42)
Other	1.31 (0.52 to 3.29)	1.24 (0.48 to 3.16)
Connective tissue diseases	0.96 (0.41 to 2.22)	0.95 (0.37 to 2.41)
Rheumatoid arthritis	1.51 (0.81 to 2.79)	0.81 (0.4 to 1.66)
Spondyloarthritis	0.62 (0.31 to 1.2)	0.78 (0.38 to 1.58)
**Treatment** ^b^		
GC-Moderate/High^c^	6.54 (2.35 to 18.63)	8.49 (2.43 to 30.89)
GC-Any	3.33 (1.8 to 6.3)	4.55 (1.66 to 12.85)
GC-Low	2.81 (1.44 to 5.56)	3.78 (1.33 to11.05)
Colchicine	1.11 (0.49 to 2.49)	2.15 (0.88 to 5.34)
Rituximab	1.94 (0.49 to 7.35)	1.62 (0.38 to 6.73)
Immunosuppressives	1.14 (0.42 to 3.01)	1.59 (0.53 to 4.77)
Hydroxychloroquine	1.82 (0.92 to 3.59)	1.43 (0.67 to 3.07)
Other bDMARDs^d^	0.63 (0.29 to 1.36)	0.9 (0.39 to 2.03)
No treatment	0.46 (0.16 to 1.29)	0.59 (0.19 to 1.76)
Other csDMARDs^e^	0.97 (0.54 to 1.76)	0.36 (0.17 to 0.75)
**Comorbidity** ^f^		
Chronic kidney disease	11.19 (2.06 to 85.38)	12.87 (2.25 to 103.5)
Cerebrovascular accident	6.41 (1.33 to 35.06)	4.94 (0.96 to 29.12)
Obesity	3.8 (1.19 to 12.29)	3.7 (1.01 to 13.87)
Cardiovascular disease	4.34 (1.63 to 11.74)	2.49 (0.81 to 7.83)
Pulmonary disease	2.55 (1.14 to 5.68)	2.66 (1.08 to 6.61)
AID	0.99 (0.22 to 4.17)	1.79 (0.38 to 8.39)
Hypertension	2.83 (1.48 to 5.46)	1.22 (0.55 to 2.71)
Diabetes	1.94 (0.71 to 5.19)	1.15 (0.4 to 3.28)
Malignancy	1.32 (0.31 to 5.27)	1.09 (0.26 to 4.48)
Other	0.66 (0.15 to 2.85)	0.89 (0.17 to 4.68)
None	0.26 (0.14 to 0.49)	0.41 (0.2 to 0.8)

AID, additional immune disorder; bDMARDs, biologic DMARDs; csDMARDs, conventional synthetic DMARDs; DMARDs, disease-modifying antirheumatic drugs; GC, glucocorticoid; IRD, inflammatory rheumatic disease.

ORs were calculated using proportional odds models for the 4-level ordinal outcome categories.

a Adjusted by age, sex and comorbidity.

b Adjusted by age, sex, comorbidity and use of solo vs combined treatment.

c Moderate/high glucocorticoid dose was defined as a daily dose of≥7.5 mg prednisolone.

d Other bDMARDs included tumor necrosis factor inhibitors, abatacept, anakinra, secukinumab and tocilizumab.

e csDMARDs included methotrexate, sulfasalazine, leflunomide, immunosuppressives included mycophenolate mofetil, tacrolimus, azathioprine.

f Adjusted by age, sex and smoking status, 19 patients was excluded from the analysis due to missing smoking data.

Among the IRD treatments, glucocorticoids, especially with moderate to high doses were associated with increased risk of a worse outcome **(** **Table 3** **).** The use of csDMARDs other than HQ were associated with a reduced risk of a worse outcome compared with patients not using csDMARDs. Colchicine, bDMARDs, and rituximab use were not significantly associated with a worse outcome of COVID-19. Comorbidities with the highest relative risk for worse outcomes were chronic kidney disease, pulmonary disease and obesity. Having no comorbidities was associated with a decreased risk of a worse outcome **(** **Table 3** **).**

The results were similar when the data was analyzed with a 5-level ordinal outcome, where ICU and death were classified as separate categories **(** **Supplementary Figure S5** **).**

Course of rheumatic diseases during COVID-19 are provided in the **Supplementary Material**.

## Discussion

This study showed that one third of IRD patients required oxygen support, 13% were treated in the ICU and the overall mortality rate was 10% due to COVID-19. We found that glucocorticoid use, having chronic kidney disease, pulmonary disease and obesity are associated with a worse outcome and the risk of a worse outcome is decreased in patients without comorbidities and in patients treated with csDMARDs. None of the IRD diagnoses by itself was associated with a worse outcome.

Since excessive inflammatory response is known to be associated with severe COVID-19, it is of particular interest to find out whether a specific IRD diagnosis influences COVID-19 outcome. Two studies with a relatively small number of patients found no association between IRD diagnoses and a worse outcome (11, 12) and two other studies showed a higher risk for severe COVID-19 among patients with diagnoses other than inflammatory arthritis such as connective tissue diseases, vasculitis and sarcoidosis (13, 14). In our cohort, none of the relative risk estimates was sufficiently precise to make strong conclusions about the associations between IRD diagnoses and a worse outcome. However, after adjusting by age, sex and comorbidity status, the point estimates for FMF and BS indicated the highest relative risk compared to the rest of the study population.

The GRA and Surveillance Epidemiology of Coronavirus Under Research Exclusion for Inflammatory Bowel Disease registries were in agreement that glucocorticoids increases the risk of severe disease (7, 15, 16). Different from the GRA registry which found an increased risk for severe disease with only a dose of ≥10 mg prednisolone use, any dose of glucocorticoids was associated with a worse outcome in our cohort. The odds ratio for a worse outcome was the highest with moderate to high dose glucocorticoid use. Although glucocorticoids may have anti-inflammatory properties that help to control severe symptoms of hyperinflammatory phase, they may enhance viral replication during the early infection phase by impairing host-defense.

Epidemiologic surveys assessing the prevalence and severity of COVID-19 among patients treated with bDMARDs and targeted synthetic DMARDs (tsDMARDs) did not show an increased risk attributable to these agents (17–20). The GRA registry showed that using bDMARDs or tsDMARDs is not associated with hospitalization and death (7, 16), moreover, patients treated with TNFis had a 60% decreased risk of hospitalization (7). Another finding of our study was that patients on csDMARDs had a reduced risk of a worse outcome. Interestingly, an observational multicenter study including 1641 IRD patients showed an increased prevalence of COVID-19 among IRD patients not on csDMARDs and suggested a protective role of csDMARDs (21). But, this study did not elaborate on the severity of COVID-19 infection among patients who used csDMARDs and those who did not. On the other hand, in the latest report from the GRA registry sulphasalazine monotherapy was associated with a higher mortality than methotrexate (16). However, physicians may have a tendency to treat IRD patients with comorbidities associated with severe COVID-19 with sulphasalazine, as the authors indicated. HQ has failed in randomized controlled and large-scale observational studies, and our results consistently showed no beneficial effect of HQ on the outcome of COVID-19 (22–25). Colchicine, another promising drug due to its anti-inflammatory properties (26), was not associated with a reduced risk for severe COVID-19.

In line with previous studies (7, 11, 13, 16), our IRD patients with comorbidities had an increased risk for a severe outcome, similar to that observed in the general population (2). The study size was not sufficient, on the other hand, to analyze comorbidities of special interest for IRDs such as interstitial lung disease or amyloidosis.

The most important limitation of our study is a possible bias toward the inclusion of more severe patients since these were patients whose COVID-19 were known to their rheumatologists. This is reflected by a high mortality rate (16/165, 10%) and a relatively small proportion of patients that recovered on outpatient management. Patients with mild disease who did not require hospitalization were possibly less likely to be captured, therefore missing from this registry. However, the GRA registry also reported a mortality rate of 10.5% among the 3729 patients although the proportion of hospitalized patients was lower in the GRA registry than in our registry (49% vs 85.5%) (16). This finding may reflect differences between countries in hospitalization policies during COVID-19 pandemic, as well as the selection bias that exists in both registries. Our other limitation was that we did not have a non-IRD comparator group in this registry. This limits our inference to within-group comparisons and identifies subgroups at relatively higher risk among patients with IRDs but cannot quantify their risk relative to the general COVID-19 patient population. On the other hand, we were able to collect sizeable single-country data with relatively homogenous IRD-treatment patterns and centrally-regulated, homogenous COVID-19 management guidelines which also includes patients with FMF and BS that are much rare outside Turkey. This may facilitate identification of disease or treatment-specific risks in comparison to more heterogeneous multi-national registries. Using an ordinal outcome that reflects a graded disease severity including the need for inpatient supplemental oxygen, ICU admission and death is another strength of this study. The most commonly used dichotomous outcome, hospitalization, includes not only an element of true disease severity but also an element of provider-perceived risk, which may obscure or distort true relationships between IRD characteristics and disease severity and may also explain some of the discrepancies between our results and others.

In conclusion our study showed that having comorbidities and glucocorticoid use were significantly associated with a worse COVID-19 outcome in IRD patients. Treatment with bDMARDs does not seem to affect COVID-19 outcome while csDMARDs may have a protective effect against the development of severe disease.

## Data Availability Statement

The original contributions presented in the study are included in the article/**Supplementary Material**. Further inquiries can be directed to the corresponding author.

## Ethics Statement

The studies involving human participants were reviewed and approved by the Ministry of Health and Cerrahpasa Medical School, Istanbul University-Cerrahpasa (83045809-604.01.02). Written informed consent for participation was not required for this study in accordance with the national legislation and the institutional requirements.

## Collaboration Group Information

Turkish Society for Rheumatology COVID-19 Registry Investigators: **Ayse Cefle, MD**, Kocaeli University, Faculty of Medicine, Department of Internal Medicine, Division of Rheumatology, Kocaeli, Turkey; **Ali Karakas, MD**, Dokuz Eylül University, Faculty of Medicine, Department of Internal Medicine, Division of Rheumatology, Izmir, Turkey; **Derya Kaskari, MD**, Başkent University Istanbul Hospital, Department of Internal Medicine, Division of Rheumatology, Istanbul, Turkey; **Samet Karahan, MD**, Kayseri City Training and Research Hospital, Department of Rheumatology, Kayseri, Turkey; **Dilek Tezcan, MD**, Selçuk University, Faculty of Medicine, Department of Internal Medicine, Division of Rheumatology Konya, Turkey; **Abdurrahman Tufan, MD**, Gazi University, Faculty of Medicine, Department of Internal Medicine, Division of Rheumatology, Ankara, Turkey; **Ayse Ayan**, Antalya Training and Research Hospital, Department of Rheumatology, Antalya, Turkey; **Levent Kılıc, MD**, Hacettepe University, Faculty of Medicine, Department of Internal Medicine, Division of Rheumatology, Ankara, Turkey; **Salim Donmez, MD**, Private Practice, Yalova, Turkey; **Mustafa Erdogan, MD**, Başakşehir Çam and Sakura City Hospital, Department of Rheumatology, Istanbul, Turkey; **Veli Yazisiz, MD**, Akdeniz University, Faculty of Medicine, Department of Internal Medicine, Division of Rheumatology, Antalya, Turkey; **Edip Gokalp Gok, MD**, Health Sciences University, Mehmet Akif Inan Training and Research Hospital, Department of Rheumatology, Şanlıurfa, Turkey; **Ahmet Eftal Yucel, MD**, Başkent University, Faculty of Medicine, Department of Internal Medicine, Division of Rheumatology, Ankara, Turkey; **Elif Dincses Nas, MD**, Istanbul Medeniyet University, Göztepe Training and Research Hospital, Department of Rheumatology, Istanbul, Turkey; **Gezmiş Kimyon, MD**, Hatay Mustafa Kemal University, Faculty of Medicine, Department of Internal Medicine, Division of Rheumatology, Hatay, Turkey; **Gunay Sahin Dalgic, MD**, Başkent University, Faculty of Medicine, Department of Internal Medicine, Division of Rheumatology, Ankara, Turkey; **Hakan Erdem, MD**, Medicana International Ankara Hospital, Department of Rheumatology, Ankara, Turkey; **Kerem Yigit Abacar, MD**, Marmara University, Faculty of Medicine, Department of Internal Medicine, Division of Rheumatology, Istanbul, Turkey; MD, **Ridvan Mercan, MD**, Namık Kemal University, Faculty of Medicine, Department of Internal Medicine, Division of Rheumatology, Tekirdağ, Turkey; **Omer Karadag, MD**, Hacettepe University, Faculty of Medicine, Department of Internal Medicine, Division of Rheumatology, Ankara, Turkey; **Onay Gercik, MD**, Health Sciences University, Izmir Tepecik Training and Research Hospital, Department of Rheumatology, Izmir, Turkey; **Suleyman Ozbek, MD**, Çukurova University, Faculty of Medicine, Department of Internal Medicine, Division of Rheumatology, Adana, Turkey; **Sebnem Gider, MD**, Medicine Hospital, Istanbul, Turkey; **Semih Gulle, MD**, Dokuz Eylül University, Faculty of Medicine, Department of Internal Medicine, Division of Rheumatology, Izmir, Turkey; **Sibel Osken, MD**, Health Sciences University, Fatih Sultan Mehmet Training and Research Hospital, Department of Rheumatology, Istanbul, Turkey; **Sedat Kiraz, MD**, Hacettepe University, Faculty of Medicine, Department of Internal Medicine, Division of Rheumatology, Ankara, Turkey; **Timucin Kasifoglu, MD**, Eskişehir Osmangazi University, Faculty of Medicine, Department of Internal Medicine, Division of Rheumatology, Eskişehir, Turkey; **Fatma Alibaz-Oner, MD**, Marmara University, Faculty of Medicine, Department of Internal Medicine, Division of Rheumatology, Istanbul, Turkey; **Izzet Fresko, MD**, Istanbul University−Cerrahpaşa, Cerrahpaşa Faculty of Medicine, Department of Internal Medicine, Division of Rheumatology, Istanbul, Turkey; **Ali Akdogan, MD**, Hacettepe University, Faculty of Medicine, Department of Internal Medicine, Division of Rheumatology, Ankara, Turkey; **Neslihan Yilmaz, MD**, Demiroğlu Bilim University, Faculty of Medicine, Department of Internal Medicine, Division of Rheumatology, Istanbul, Turkey.

## Author Contributions

SE: Visualization, Investigation, Writing - Original Draft. KT: Formal analysis, Writing - Original Draft. HB: Investigation, Writing - review & editing. CB: Conceptualization, Methodology, Investigation, Writing - review & editing. BY: Investigation, Writing - review & editing. SA: Conceptualization, Methodology, Investigation, Writing - review & editing. OP: Investigation, Writing - review & editing. CA: Investigation, Writing - review & editing. DT: Investigation, Writing - review & editing. ES: Investigation, Writing - review & editing. TY-I: Investigation, Writing - review & editing. NA-K: Investigation, Writing - review & editing. EB: Investigation, Writing - review & editing. ET: Investigation, Writing - review & editing. SC: Investigation, Writing - review & editing. EB: Investigation, Writing - review & editing. SK: Conceptualization Methodology, Investigation, Writing - review & editing. UK: Conceptualization Methodology, Investigation, Writing - review & editing. OI: Investigation, Writing - review & editing. SU: Investigation, Writing - review & editing. HO: Investigation, Writing - review & editing. VH: Conceptualization Methodology Supervision, Writing- Reviewing and Editing. GH: Conceptualization, Methodology, Writing - Original Draft, Supervision. All authors contributed to the article and approved the submitted version.

## Conflict of Interest

KT has served as a speaker for Gilead. GH has received grant/research support from Celgene and has served as a speaker for AbbVie, Celgene, Novartis, and UCB Pharma.

The remaining authors declare that the research was conducted in the absence of any commercial or financial relationships that could be construed as a potential conflict of interest.

## References

[1] Wu Z McGoogan JM. Characteristics of and Important Lessons From the Coronavirus Disease 2019 (COVID-19) Outbreak in China: Summary of a Report of 72314 Cases From the Chinese Center for Disease Control and Prevention. Jama (2020) 323:1239–42. 10.1001/jama.2020.2648 32091533

[2] Jain V Yuan JM. Predictive symptoms and comorbidities for severe COVID-19 and intensive care unit admission: a systematic review and meta-analysis. Int J Public Health (2020) 65:533–46. 10.1007/s00038-020-01390-7 PMC7246302 32451563

[3] Kastritis E Kitas GD Vassilopoulos D Giannopoulos G Dimopoulos MA Sfikakis PP. Systemic autoimmune diseases, anti-rheumatic therapies, COVID-19 infection risk and patient outcomes. Rheumatol Int (2020) 40:1353–60. 10.1007/s00296-020-04629-x PMC7353833 32654078

[4] Zhong J Shen G Yang H Huang A Chen X Dong L. COVID-19 in patients with rheumatic disease in Hubei province, China: a multicentre retrospective observational study. Lancet Rheumatol (2020) 2:e557–564. 10.1016/S2665-9913(20)30227-7 PMC7333992 32838309

[5] Ye C Cai S Shen G Guan H Zhou L Hu Y. Clinical features of rheumatic patients infected with COVID-19 in Wuhan, China. Arthritis Rheumatol (2020) 79:1007–13. 10.1007/s10067-020-05160-x PMC7295865 32444415

[6] Sarzi-Puttini P Giorgi V Sirotti S Marotto D Ardizzone S Rizzardini G. COVID-19, cytokines and immunosuppression: what can we learn from severe acute respiratory syndrome? Clin Exp Rheumatol (2020) 38:337–42. 32202240

[7] Gianfrancesco M Hyrich KL Al-Adely S Carmona L Danila MI Gossec L. Characteristics associated with hospitalisation for COVID-19 in people with rheumatic disease: data from the COVID-19 Global Rheumatology Alliance physician-reported registry. Ann Rheum Dis (2020) 79:859–66. 10.1136/annrheumdis-2020-217871 PMC7299648 32471903

[8] World Health Organization. WHO R&D Blueprint novel Coronavirus COVID-19 Therapeutic Trial Synopsis. (2020). Available at: https://www.who.int/blueprint/priority-diseases/key-action/COVID-19_Treatment_Trial_Design_Master_Protocol_synopsis_Final_18022020.pdf.

[9] Harris PA Taylor R Thielke R Payne J Gonzalez N Conde JG. Research electronic data capture (REDCap)–a metadata-driven methodology and workflow process for providing translational research informatics support. J BioMed Inform (2009) 42:377–81. 10.1016/j.jbi.2008.08.010 PMC2700030 18929686

[10] Textor J Hardt J Knuppel S. DAGitty: a graphical tool for analyzing causal diagrams. Epidemiology (2011) 22:745. 10.1097/EDE.0b013e318225c2be 21811114

[11] Fredi M Cavazzana I Moschetti L Andreoli L Franceschini F Brescia Rheumatology COVID-19 Study Group. COVID-19 in patients with rheumatic diseases in northern Italy: a single-centre observational and case-control study. Lancet Rheumatol (2020) 2:e549–56. 10.1016/S2665-9913(20)30169-7 PMC7302769 32838307

[12] Montero F Martinez-Barrio J Serrano-Benavente B Gonzalez T Rivera J Molina Collada J. Coronavirus disease 2019 (COVID-19) in autoimmune and inflammatory conditions: clinical characteristics of poor outcomes. Rheumatol Int (2020) 40:1593–8. 10.1007/s00296-020-04676-4 PMC7425254 32794113

[13] Pablos JL Galindo M Carmona L Lledo A Retuerto M Blanco R. Clinical outcomes of hospitalised patients with COVID-19 and chronic inflammatory and autoimmune rheumatic diseases: a multicentric matched cohort study. Ann Rheum Dis (2020) 79:1544–9. 10.1136/annrheumdis-2020-218296 32796045

[14] Freites Nunez DD Leon L Mucientes A Rodriguez-Rodriguez L Font Urgelles J Madrid Garcia A. Risk factors for hospital admissions related to COVID-19 in patients with autoimmune inflammatory rheumatic diseases. Ann Rheum Dis (2020) 79:1393–9. 10.1136/annrheumdis-2020-217984 32769150

[15] Brenner EJ Ungaro RC Gearry RB Kaplan GG Kissous-Hunt M Lewis JD. Corticosteroids, But Not TNF Antagonists, Are Associated With Adverse COVID-19 Outcomes in Patients With Inflammatory Bowel Diseases: Results From an International Registry. Gastroenterology (2020) 59:481–491.e3. 10.1053/j.gastro.2020.05.032 PMC7233252 32425234

[16] Strangfeld A Schäfer M Gianfrancesco MA Lawson-Tovey S Liew JW Ljung L. Factors associated with COVID-19-related death in people with rheumatic diseases: results from the COVID-19 Global Rheumatology Alliance physician-reported registry. Ann Rheum Dis (2021). 10.1136/annrheumdis-2020-219498 PMC7843211 33504483

[17] Quartuccio L Valent F Pasut E Tascini C De Vita S. Prevalence of COVID-19 among patients with chronic inflammatory rheumatic diseases treated with biologic agents or small molecules: A population-based study in the first two months of COVID-19 outbreak in Italy. Joint Bone Spine (2020) 87:439–43. 10.1016/j.jbspin.2020.05.003 PMC7239017 32445935

[18] Favalli EG Monti S Ingegnoli F Balduzzi S Caporali R Montecucco C. Incidence of COVID-19 in patients with rheumatic diseases treated with targeted immunosuppressive drugs: what can we learn from observational data? Arthritis Rheumatol (2020) 72:1600–6. 10.1002/art.41388 PMC7300434 32506699

[19] Salvarani C Bajocchi G Mancuso P Galli E Muratore F Boiardi L. Susceptibility and severity of COVID-19 in patients treated with bDMARDS and tsDMARDs: a population-based study. Ann Rheum Dis (2020) 79:986–8. 10.1136/annrheumdis-2020-217903 32467245

[20] Michelena X Borrell H Lopez-Corbeto M Lopez-Lasanta M Moreno E Pascual-Pastor M. Incidence of COVID-19 in a cohort of adult and paediatric patients with rheumatic diseases treated with targeted biologic and synthetic disease-modifying anti-rheumatic drugs. Semin Arthritis Rheumatol (2020) 50:564–70. 10.1016/j.semarthrit.2020.05.001 PMC7229730 32425260

[21] Ferri C Giuggioli D Raimondo V L’Andolina M Tavoni A Cecchetti R. COVID-19 and rheumatic autoimmune systemic diseases: report of a large Italian patients series. Clin Rheumatol (2020) 39:3195–204. 10.1007/s10067-020-05334-7 PMC7450255 32852623

[22] Geleris J Sun Y Platt J Zucker J Baldwin M Hripcsak G. Observational Study of Hydroxychloroquine in Hospitalized Patients with Covid-19. N Engl J Med (2020) 382:2411–8. 10.1056/NEJMoa2012410 PMC7224609 32379955

[23] Rosenberg ES Dufort EM Udo T Wilberschied LA Kumar J Tesoriero J. Association of Treatment With Hydroxychloroquine or Azithromycin With In-Hospital Mortality in Patients With COVID-19 in New York State. Jama (2020) 323:2493–502. 10.1001/jama.2020.8630 PMC7215635 32392282

[24] Tang W Cao Z Han M Wang Z Chen J Sun W. Hydroxychloroquine in patients with mainly mild to moderate coronavirus disease 2019: open label, randomised controlled trial. BMJ (2020) 369:m1849. 10.1136/bmj.m1849 32409561 PMC7221473

[25] Cavalcanti AB Zampieri FG Rosa RG Azevedo LCP Veiga VC Avezum A. Hydroxychloroquine with or without Azithromycin in Mild-to-Moderate Covid-19. N Engl J Med (2020) 383:2041–52. 10.1056/NEJMoa2019014 PMC7397242 32706953

[26] Schlesinger N Firestein BL Brunetti L. Colchicine in COVID-19: an Old Drug, New Use. Curr Pharmacol Rep (2020). 10.1007/s40495-020-00225-6 PMC7367785 32837853

