# Supplementary material for: COVID-19 Among Patients With Inflammatory Rheumatic Diseases
Source: Front Immunol. 2021 Apr 16;12:651715. doi: 10.3389/fimmu.2021.651715 (PMC8086428; doi:10.3389/fimmu.2021.651715)
Supplement: Supplementary file 1 [file DataSheet_1.pdf]

Supplementary Material

Supplementary Methods. Treatment protocols for COVID-19 in Turkey

The Turkish Ministry of Health (TMoH) issued a COVID-19 guideline for healthcare providers following the first confirmed COVID-19 case on March 11, 2020. This has been updated as new data became available.

According to the TMoH guideline revised on April 14, all probable/confirmed cases were to be treated with HQ (400 mg twice on day 1, followed by 200 mg twice a day on days 2–5), after normal corrected QT (QTc) interval was confirmed. Azithromycin could be added in hospitalized patients who did not have risk factors associated with prolonged QTc interval such as older age, hypertension or hypokalemia, with daily ECG monitoring. Favipiravir was initiated in cases with severe pneumonia and in those with progressive symptoms. Favipiravir dose was 1600 mg twice on day 1, followed by 600 mg twice a day on days 2-5. Oseltamivir was used during the flu season if favipiravir was not initiated. In case of cytokine storm tocilizumab or anakinra were recommended. Anticoagulant prophylaxis was given to all hospitalized patients. The main differences between this revised guideline and the previous version were expanded indications for favipiravir and restrictions on the use of azithromycin-HQ combination due to concerns about cardiotoxicity. Favipiravir became the widely used antiviral agent for COVID-19 outpatients at the beginning of August 2020 in Turkey, but was spared for only severe patients at the time of this study.

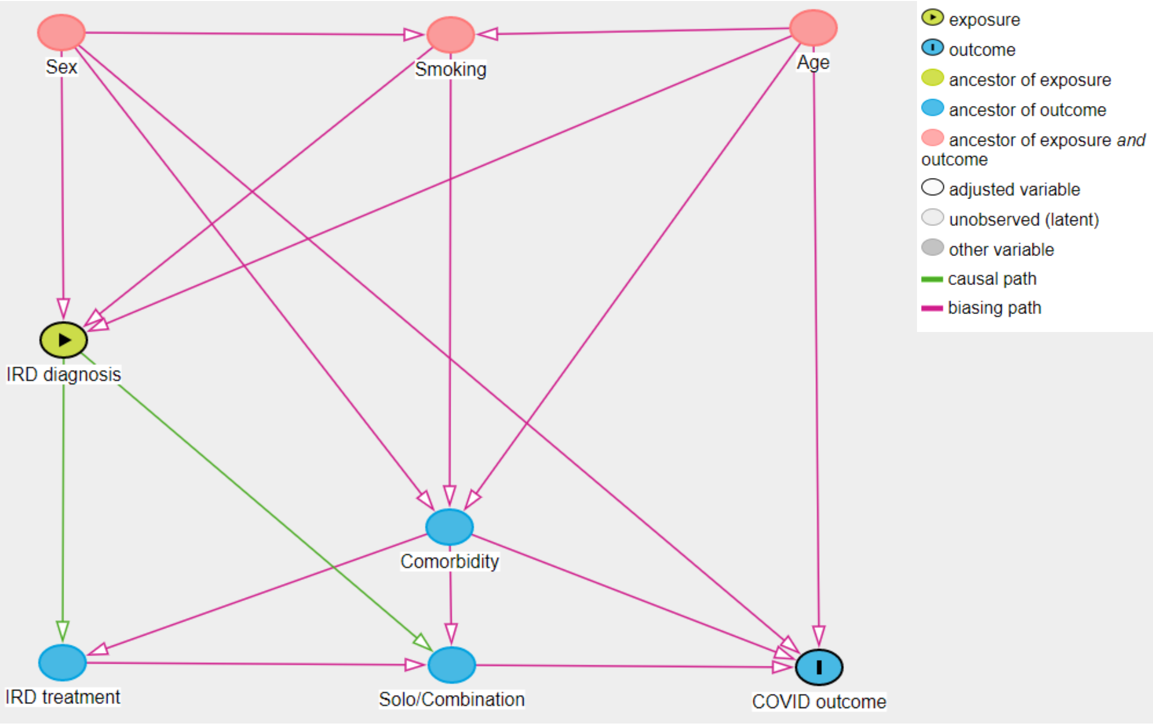

Supplementary Figure S1. Directed acyclic graph depicting causal assumptions for the effect of IRD diagnosis on COVID outcome. In order to estimate the unbiased total effect of IRD treatment on COVID outcome, two minimum sufficient sets of adjustment variables can be identified from this model, (i) age, comorbidity, sex or (ii) age, sex, smoking. Prepared using DAGitty v. 3.0 (www.dagitty.net).

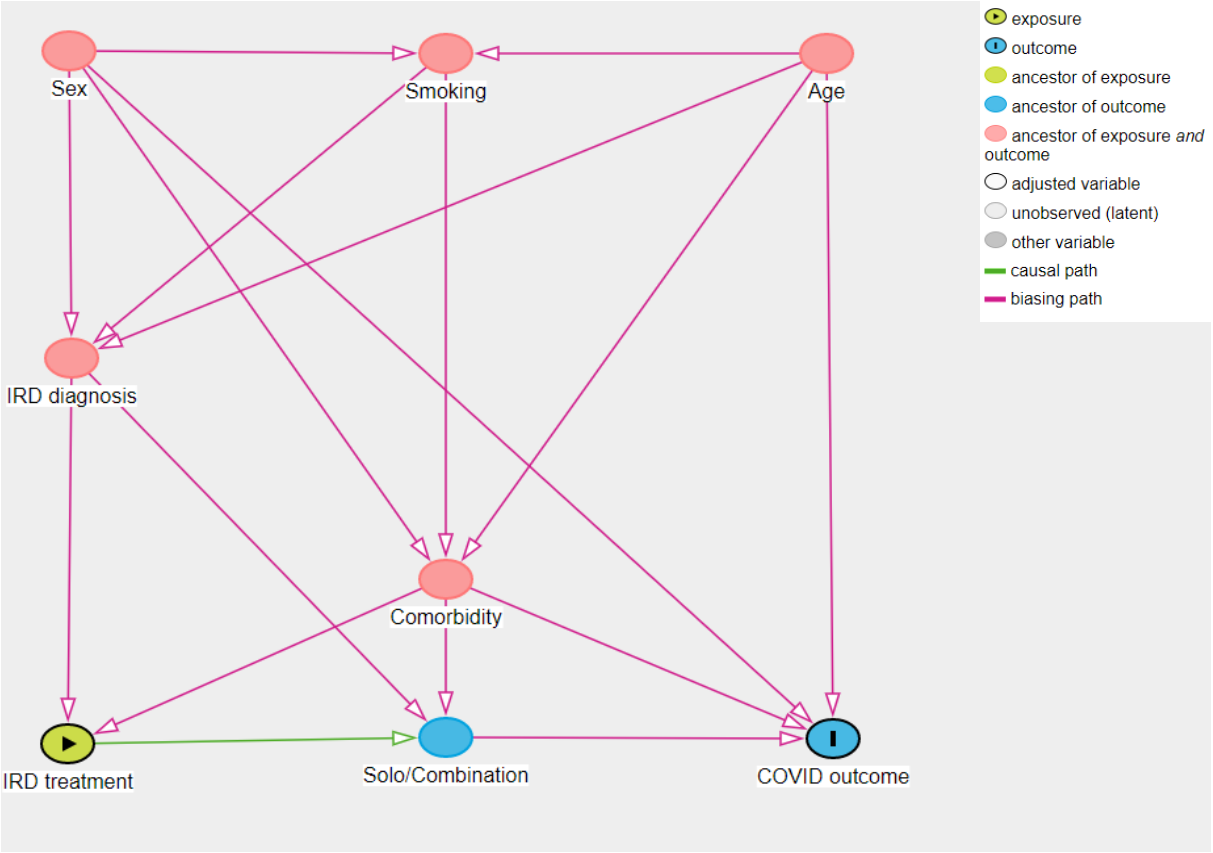

Supplementary Figure S2. Directed acyclic graph depicting causal assumptions for the effect of IRD treatment on COVID outcome. In order to estimate the unbiased direct effect of IRD treatment on COVID outcome, two minimum sufficient sets of adjustment variables can be identified from this model, (i) Age, comorbidity, sex, combination treatment, or (ii) comorbidity, inflammatory rheumatic disease (IRD) diagnosis, combination treatment. Prepared using DAGitty v. 3.0 (www.dagitty.net).

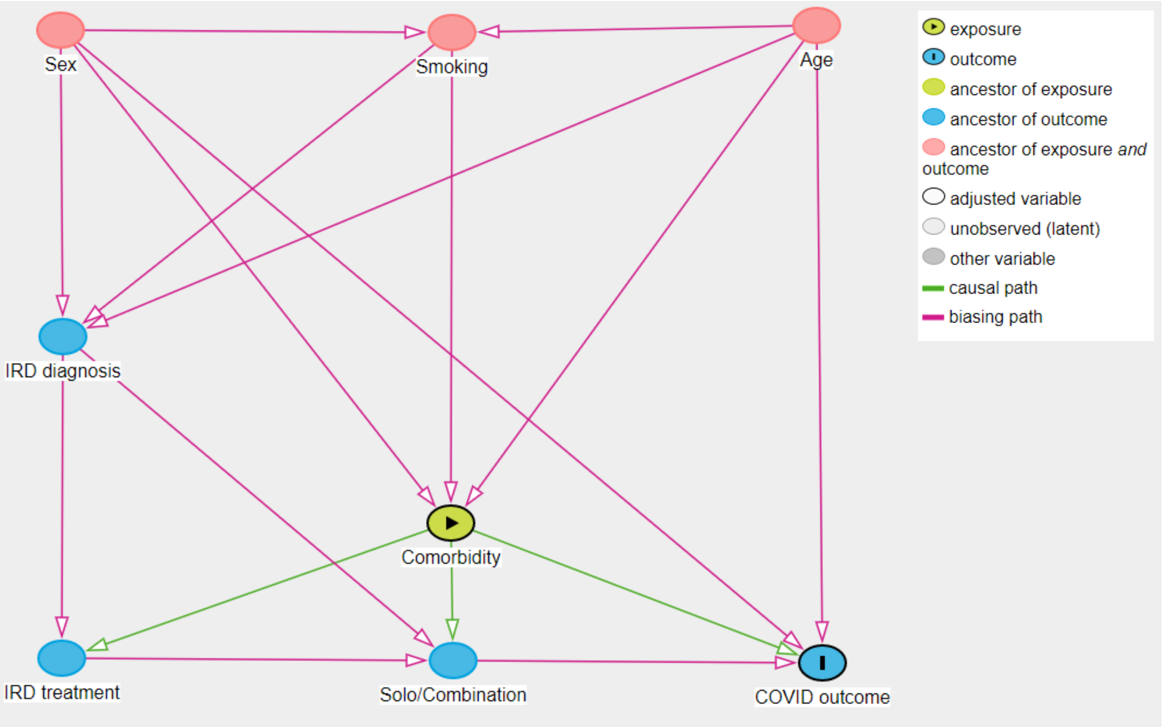

Supplementary Figure S3. Directed acyclic graph depicting causal assumptions for the effect of comorbidity on COVID outcome. In order to estimate the unbiased total effect of comorbidity on COVID outcome, two minimum sufficient sets of adjustment variables can be identified from this model, (i) age, IRD diagnosis, sex or (ii) age, sex, smoking. Prepared using DAGitty v. 3.0 (www.dagitty.net).

Supplementary Table S1. IRD diagnoses and IRD treatments that were used at the time of COVID-19 diagnosis

**Characteristics**	**n (%)**
Diagnosis[a]	
Rheumatoid arthritis	60 (36)
Spondyloarthritis	
Axial SpA (including AS)	29 (18)
Psoriatic arthritis	10 (6)
Undifferentiated SpA	3 (1.8)
Connective tissue disease	
Systemic lupus erythematosus	13 (8)
Sjögren's syndrome	8 (5)
Mixed connective tissue disease	1
Undifferentiated connective tissue disease	1
Systemic sclerosis	1
Antiphospholipid syndrome	5 (3)
Familial Mediterranean fever	14
Behçet syndrome	15 (9)
ANCA-associated vasculitis	3 (1.8)
Polymyalgia rheumatica	2 (1)
Juvenile idiopathic arthritis	2 (1)
Gout	3 (1.8)
Adult onset Still's disease	3 (1.8)
Takayasu arteritis	1
Henoch Schonlein purpura	1
Goodpasture syndrome	1
Sarcoidosis	1
Undifferentiated arthritis	1
Treatment	

NSAIDs, n/N, (%)	
Yes and continued	7/163 (4)
Yes and discontinued	15/163 (9)
No	142/163 (87)
COX-2 inhibitors, n/N, (%)	
Yes and discontinued	3/163 (2)
No	160/163 (98)
Prednisolone[b], n (%)	
None	97 (59)
<7.5 mg/day	49 (30)
≥7.5 mg/day	18 (11)
Colchicine	25 (15)
Hydroxychloroquine	40 (24)
csDMARDs	
Methotrexate	37 (22)
Leflunomide	23 (14)
Sulfasalazine	19 (11.5)
Immunosuppressives	
Azathioprine	10 (6)
Mycophenolate mofetil	5 (3)
Tacrolimus	1
bDMARDs	
Tumor necrosis factor inhibitors	22 (13)
Rituximab	7 (4)
Tocilizumab	2 (1)
Secukinumab	3 (2)
Anakinra	1
Abatacept	1
ACE inhibitors/ARBs, n/N (%)	

Yes and continued	16/164 (10)
Yes and discontinued	9/164 (5.5)

ACE: angiotensin-converting enzyme; ARBs: angiotensin II receptor blockers; AS: ankylosing spondylitis; bDMARD: biologic DMARDs; COX-2: cyclooxygenase-2; csDMARDs: conventional synthetic DMARDs; DMARDs: disease-modifying antirheumatic drugs; IRD: inflammatory rheumatic disease; NSAIDs: non-steroidal anti-inflammatory drugs, SpA: spondyloarthritis;
[a] IRD diagnoses are non-mutually-exclusive.
[b] Prednisolone dose was unknown in 1 patient.

Supplementary Results. Routes of transmission of COVID-19 and COVID-19 diagnosis among household members

Exposure to Covid-19

A possible route of transmission was indicated for 68 of the patients. Among these, the suspected route of COVID-19 transmission was close contact with a COVID-19 patient in 48/68 (44%) patients, visit to a high-risk country in 3 patients, visiting a COVID-19 referral hospital in 16/68 (15%) and close contact with a family member working at a hospital in 1 patient.

COVID-19 among household members

The number of household members was available for 95 patients. Seven of them -were living alone. Among the remaining 88 patients, 38 (43%) did not have a household member diagnosed with COVID-19. Overall, 97 (43%) of the 226 household members were diagnosed with COVID-19.

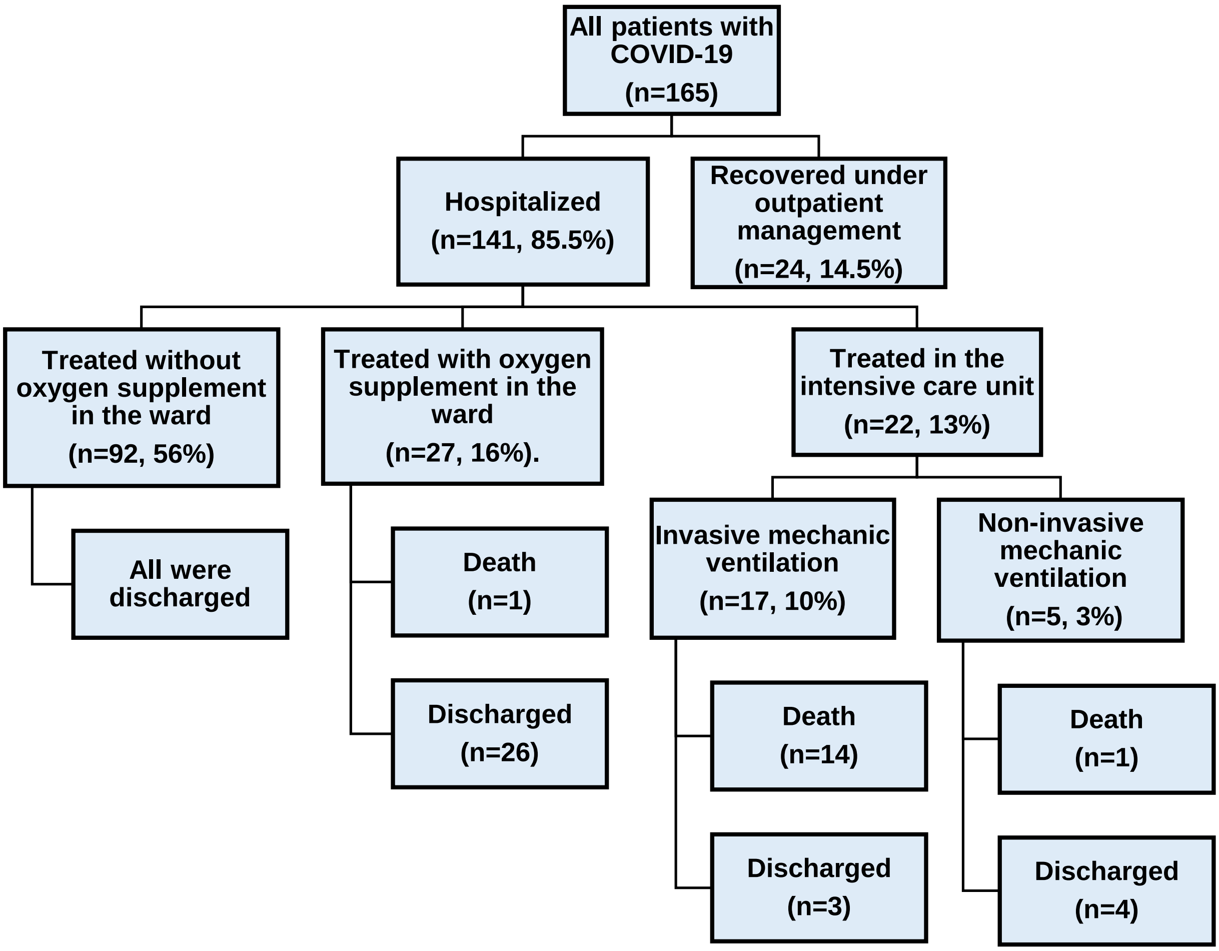

Supplementary Figure S4: Flow diagram of the outcome of COVID-19 in patients with inflammatory rheumatic diseases

Supplementary Table S2. Characteristics of patients who needed ICU admission or those who died

Gender	Age (years)	IRD diagnosis	IRD treatment	Comorbidity	ICU admission	Outcome
Female	35	SLE	HQ, MMF, PRED 20 mg/day	CKD	IMV	Died
Female	51	RA	HQ, RTX, PRED 7.5 mg/day	HT, Obesity, CKD	IMV	Died
Female	32	SLE	HQ, PRED 5 mg/day	CVA	IMV	Died
Female	72	PMR	PRED 7.5 mg/day	None	IMV	Died
Female	71	RA	HQ, PRED 5 mg/day	None	IMV	Died
Female	79	Goodpasture syndrome	PRED 40 mg/day	None	IMV	Died
Male	69	RA	SAZ	HT, CVD, CKD	IMV	Died
Female	38	BS	COL	None	NIMV	Died
Female	71	RA	HQ, MTX, PRED 5 mg/day	HT	IMV	Died
Female	64	Sarcoidosis	PRED 20 mg/day	HT, DM	IMV	Died
Female	78	RA	MTX, PRED 5 mg/day	HT	IMV	Died
Female	75	PsA	MTX, LEF	HT, DM, CVD	IMV	Died
Male	60	RA	LEF, PRED 5 mg/day	COPD, CVD	IMV	Died
Male	72	RA	HQ, PRED 10 mg/day	AML	No	Died before ICU transfer
Male	70	RA	TNFi, PRED 5 mg/day	DM, CVD, CVA	IMV	Died
Female	32	FMF, SpA	AZA, COL, TNFi, PRED 10 mg/day	Crohn's disease	IMV	Died

Gender	Age (years)	IRD diagnosis	IRD treatment	Comorbidity	ICU admission	Outcome
Female	73	RA	HQ, MTX, PRED 10 mg/day	HT	IMV	Recovered
Male	38	FMF	COL	HT, Obesity	NIMV	Recovered
Male	58	BS	COL, SAZ	None	NIMV	Recovered
Male	55	BS	COL	HT, Asthma	IMV	Recovered
Female	58	Sjögren's syndrome	HQ, PRED 5 mg/day	HT, DM, ILD	NIMV	Recovered
Female	88	RA	HQ, LEF, PRED 7.5 mg/day	HT, CVD, CKD	IMV	Recovered
Female	57	RA	LEF, PRED 5 mg/day	HT, DM, Obesity	NIMV	Recovered

AML: acute myeloid leukemia; BS: Behçet syndrome; CKD: chronic kidney disease; COL: colchicine; COPD: chronic obstructive pulmonary disease; CVA: cerebrovascular accident; CVD: cardiovascular disease; DM: diabetes mellitus; FMF: familial Mediterranean fever; HQ: hydroxychloroquine; HT: hypertension; ICU: intensive care unit; ILD: interstitial lung disease; IMV: invasive mechanic ventilation; IRD: inflammatory rheumatic disease; LEF: leflunomide; MMF: mycophenolate mofetil; MTX: methotrexate; NIMV: non-invasive mechanic ventilation; PMR: polymyalgia rheumatica; PRED: prednisolone; PsA: psoriatic arthritis; RA: rheumatoid arthritis; RTX: rituximab; SAZ: sulfasalazine; SLE: systemic lupus erythematosus; SpA: spondyloarthritis; TNFi: tumor necrosis factor inhibitor

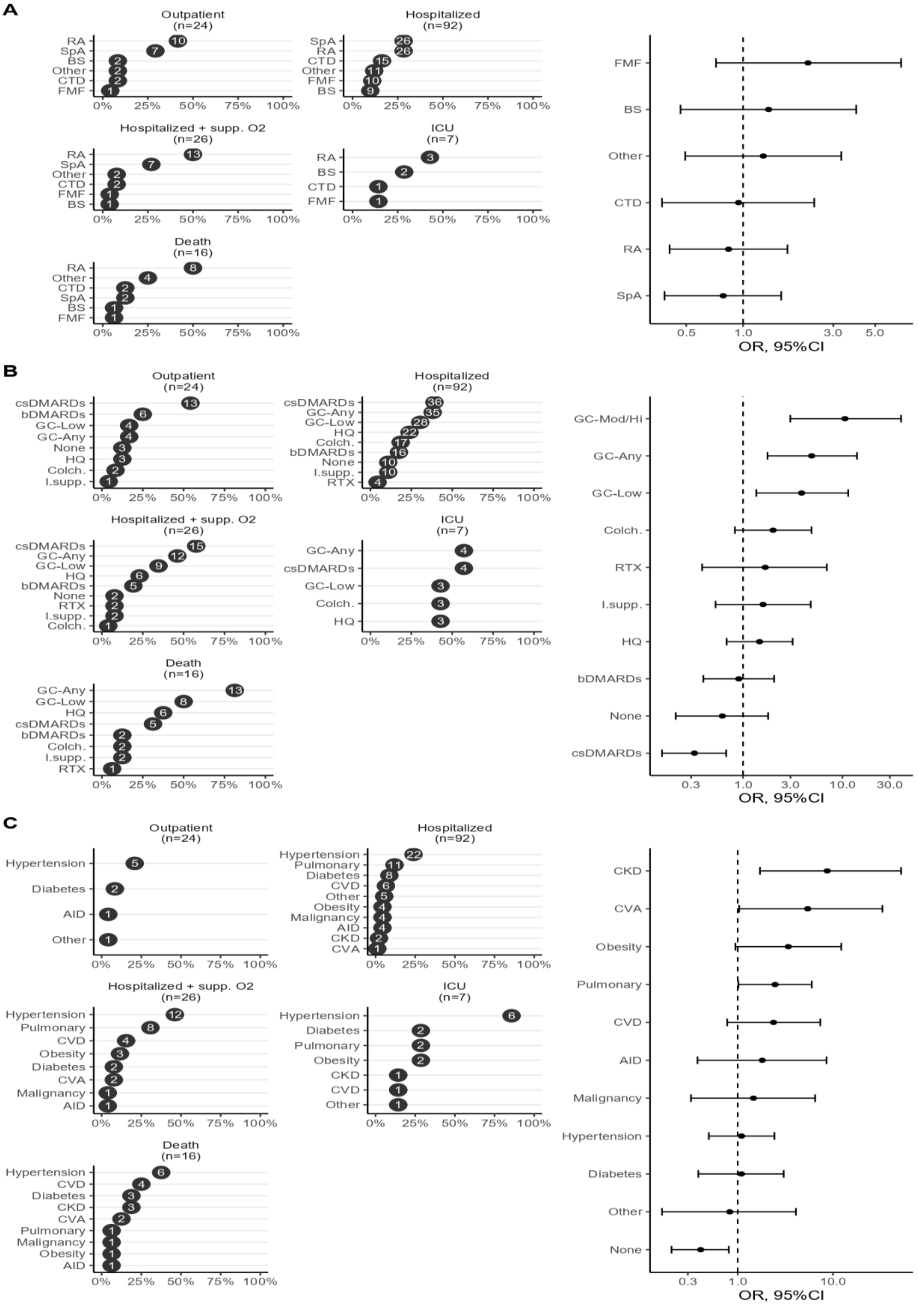

Supplementary Figure S5: Risk factors of a worse COVID-19 outcome in patients with inflammatory rheumatic diseases.

Figure illustrates the ranked proportions of IRD diagnoses (A), IRD treatments (B), and comorbidities (C) for each outcome category and the association of each with a worse outcome, analyzed using proportional odds logistic regression where the dependent variable was the 5-level ordinal outcome and the independent variable of interest was the type of IRD treatment, comorbidity or primary IRD diagnosis.

Disease outcome was classified in 5 ordinal categories; (i) outpatient management, (ii hospitalization without oxygen requirement, (iii) hospitalization with oxygen requirement, (iv) ICU admission, and (v) death.

High/moderate glucocorticoid dose (GC-Mod/Hi) was defined as a daily dose of≥7.5 mg prednisolone.

csDMARDs included methotrexate, sulfasalazine, leflunomide, immunosuppressives included mycophenolate mofetil, tacrolimus, azathioprine and bDMARDs included tumor necrosis factor inhibitors, abatacept, anakinra, secukinumab and tocilizumab.

AID: additional immune disorder; bDMARDs, biologic DMARDs; BS: Behçet syndrome; CKD: chronic kidney disease; Colc.: colchicine; csDMARDs, conventional synthetic DMARDs; CTD: connective tissue disease; CVA: cerebrovascular accident; CVD: cardiovascular disease; DMARDs, disease-modifying antirheumatic drugs; FMF: Familial Mediterranean fever; GC: glucocorticoid; ICU: intensive care unit; IRD: inflammatory rheumatic disease; I.supp.: immunosuppressives; HQ: hydroxychloroquine; RA: rheumatoid arthritis; RTX: rituximab; SpA: spondyloarthritis;
